# Chimeric Antigen Receptor T-Cells (CAR T-Cells) for Cancer Immunotherapy – Moving Target for Industry?

**DOI:** 10.1007/s11095-018-2436-z

**Published:** 2018-05-31

**Authors:** Paula Salmikangas, Niamh Kinsella, Paul Chamberlain

**Affiliations:** NDA Group, Stockholm, Sweden

**Keywords:** antigen receptor, CAR T, genetic modification, immunotherapy, T-cells

## Abstract

The first CD19 CAR T-cell products, Kymriah and Yescarta, are entering the US market and also being evaluated for marketing authorization in the EU. This breakthrough has expanded the interest and also investments towards novel chimeric antigen receptor (CAR) designs, both for hematological malignancies and solid tumors. At the same time, there is active development in moving from autologous products to allogeneic, off-the-shelf -products. New manufacturing technologies are also emerging for production of these complex genetically-modified cells and even decentralized manufacturing in hospitals is under consideration. However, the high potency of CAR T-cells is associated with toxicity and not all patients respond to the treatment. In addition, the number of patient and product variables impacting the clinical outcome is high. The race towards novel CAR T treatment options for cancer patients has begun, but without careful design of the constructs and overall understanding of the factors that impact the ultimate outcome in each case, the road towards commercial success may be long and winding. This review discusses the product- and patient-related variables that may pose challenges for the industry and developers both from the scientific and regulatory perspective.

## Introduction

Adoptive cancer immunotherapies are developed using different cell types and underlying mechanisms; however, common to all of these products is the goal to induce patient’s own immune response against the tumor cells via specific tumor cell recognition and induction of cytotoxicity. This involves specific tumor-associated antigens (TAAs), recognized by genetically modified T-cell/NK-cell receptors or chimeric antigen receptors (CARs). The first CAR T-cell products, Yescarta from Kite Pharma/Gilead and Kymriah from Novartis, were approved by the United States Food and Drug Administration (US FDA) in 2017, with prominent efficacy results [1, 2]. Both products are intended for treatment of B-cell malignancies (lymphoma and leukemia) and utilize the CD19 antigen as the TAA. CD19 is an ideal target for T-cell mediated killing due to its´ specificity; the expression is restricted to B-cells and B-cell precursors and it is not found on hematopoietic stem cells [3]. This minimizes off-target toxicity and enhances anti-tumor efficacy. Both Yescarta and Kymriah are currently under assessment for marketing authorization in the European Union (EU) [4, 5], yet for Yescarta the path has not been straight forward as major objections were raised during the assessment and the original accelerated assessment timetable was reverted to the normal review period in December 2017 [6]. In advanced development phase is also the CD19 CAR T product JCAR017 from Celgene (originally developed by Juno), for which first clinical results from the TRANSCEND trial were presented in 2017 [7]. While all these three products share the same target and also the same binding domain in the CAR construct, there are differences in the design of the signaling domains of the construct and in many aspects of the manufacturing process, including the cell population used as starting material for transduction (see Table I.) [8–10]. Other tumor antigens are also utilized in the CARs designed for hematological malignancies, for example CD20 and CD22 for B-cell malignancies, BMCA for multiple myeloma and CD123 for myeloid malignancies [11].

**Table I:**
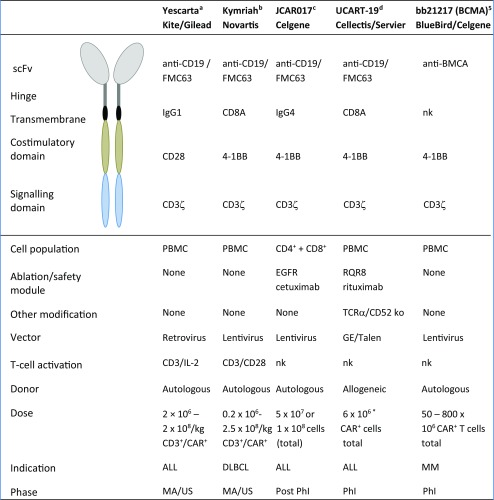
Differences in design, materials and clinical use of first approved autologous CAR T-cell products compared to other CAR T-cells (Autologous and Allogeneic) currently in clinical trials.

^a^Yescarta, US FDA assessment report [1]

^b^Kymriah, US FDA assessment report [2]

^c^JCAR017 clinical trial [10, 41]

^d^Poirot L. et al. [22] and ASH abstract 887 (2017) [23]

^e^bb21217 clinical trial [19], BlueBirdBio/Celgene press release [56] and Garrett et al. [57]

*CALM dose escalation trial, first dose results presented

nk = not known

Kymriah, Yescarta JCA017 and bb21217 have received the EU PRIME status [58]

More recently, novel tumor associated antigens (neoantigens) have come into the center of active research, especially for solid tumors [12] and also the clinical translation of novel products is strong, which can be seen e.g. from all the clinical trials (> 250) with different CAR T-cells registered into ClinicalTrials.gov. The main issue for the neoantigens is specificity, as wide expression of the antigen will usually lead also to on-target/off-tumor toxicity [12]. The CAR T approach for solid tumors also faces other challenges, for example the inhibiting tumor environment, poor access to the entire tumor tissue for CAR T-cells, etc. [12, 13]. In addition, the design of the signaling part of the CARs has evolved during the years. The first generation constructs had only one signaling domain, the cluster of differentiation 3 zeta (CD3ζ), to induce T-cell activation [14]. This approach, however, led to modest activation and to the second generation CARs additional co-stimulatory domain was included. Addition of a single CD28 or 4-1BB costimulatory domain led to improved activation and proliferation of the modified T-cells [14]. More recently it has been found that the signals transmitted by the co-stimulatory domains seem to differ and impact the ultimate T-cell composition and persistence of the CAR-expressing cells, as well as the tumor responses [15]. Clinical results are also suggesting that these highly activated T-cells could cause overly high selective pressure in the patient leading to tumor antigen escape and relapse [16]. The capability of the tumor cells to edit themselves is a challenge and the answer to this problem has been searched from dual CAR Ts, for example against CD19 and CD20 for B-cell malignancies [17]. Third generation CAR Ts involve two co-stimulatory domains (e.g. both CD28 and 4-1BB), whereas the fourth generation constructs called TRUCKs (T cells Redirected for Universal Cytokine-mediated Killing) are armored second generation CARs with additional genetic modifications to enhance anti-tumor activity, e.g. expression of cytokines [18]. In addition, the first CAR Ts with ablation/safety systems are in early clinical trials, enabling removal of the cells in case severe or life threatening adverse events jeopardize patients´ survival post administration of the cells [7, 19].

Initial CAR T developments have been based on patients’own (autologous) cells, which are collected while the patient is receiving standard cancer treatments, and then genetically modified via *ex-vivo* transduction using a product-specific vector. After transduction a carefully controlled manufacturing process is executed to expand the cells, which are then administered back to the patient. Yescarta is indicated for the treatment of adult patients with relapsed or refractory diffuse large B-cell lymphoma (R/R DLBCL) after two or more lines of systemic therapy, whereas Kymriah is indicated for the treatment of patients up to 25 years of age with B-cell precursor acute lymphoblastic leukemia (ALL) that is refractory or in second or later relapse [1, 2]. This has posed clear challenges, as the health condition and proliferation capacity of the cells to be transduced may vary significantly between patients, even leading to suboptimal cell numbers for some patients. This has moved the interest towards allogeneic (donated by other individuals), off-the-shelf products, derived from cells obtained from healthy volunteers and multiple allogeneic CAR Ts are currently in early clinical studies, including projects comparing clinical response in autologous vs. allogeneic setting using the same CAR [20].

The field of adaptive immunotherapies is fast evolving and novel technologies e.g. for genetic engineering are utilized. Gene editing (GE) is quickly moving the focus away from retro/lentiviral vectors for genetic manipulation of cells, yet the potential for off-target gene editing and corresponding safety aspects of GE are not fully solved. The products are also becoming more and more complex, having more known and unknown risks that require careful controls and patient follow-up.

## From Autologous to Allogeneic T-Cells

As can be expected, expansion and manufacturing of autologous modified T-cells from lymphocytes of heavily treated patients is not always easy and successful due to low lymphocyte counts and poor health condition of the cells. The problem is highlighted by the results of the Kymriah pivotal trial, where 9% of the enrolled subjects could not receive the product due to manufacturing failure [2]. Thus, off-the-shelf allogeneic CAR T-cells, manufactured from lymphocytes of healthy donors, seem attractive in many ways, yet there are still many issues that hamper their wider use in clinical trials.

Allogeneic cells may suffer from the human leucocyte antigen (HLA) mismatch between donor and recipient, which in worst case can lead to severe, even life threatening Graft vs. Host Disease (GvHD) [21]. Rejection could also remove the CAR-expressing cells and lead to treatment failure. To overcome this problem, HLA knock-out allogeneic CAR T-cells have been designed, e.g. the anti-CD19 UCART19 from Cellectis/Servier, where a knock-out design of the αβ T-cell receptors is expected to prevent alloreactivity [22, Table I]. According to first results of the UCART19 CALM trial, involving R/R B-ALL patients, four out of six patients with the starting dose relapsed 4–6 months post administration and one patient was reported to have probable skin GvHD, suggesting presence of partially functional HLA recognition [23].

The dose for allogeneic CAR T-cells is not as easy to define as for autologous ones, for which already a lot of information is available from multiple clinical studies. In addition, the cellular growth kinetics of healthy cells is different from cells of diseased patients and very high expansion rate of healthy cells may pose a safety risk, which needs to be considered in dose selection. For UCART19, 4 different dose levels are intended, starting with of 6 × 10^6^ total CAR+ cells, whereas for autologous CAR Ts doses in range of 10^6^–10^8^ CAR+ T-cells/kg are used. In another allogeneic CAR T trial sponsored by Cellectis (UCART123), intended to treat patients with acute myeloid leukemia (AML) and blastic plasmacytoid dendritic cell neoplasm (BPDCN), the first dosed patient died due to lethal cytokine storm and life-threatening capillary leak syndrome [24]. The trial was put on hold by FDA and a recommendation from the Data Safety Monitoring Board (DSMB) was given to lower the original dose of 625,000 CAR T + −cells/kg to 62,500 CAR-T + −cells/kg, and to reduce the conditioning cyclophosphamide dose. This case illustrates the difficulty of estimating an acceptable starting dose when using CAR T-cells that can undergo relatively strong antigen-driven proliferation, allied to potential for on-target/off-tumor toxicity for an antigen (CD123) that is expressed on normal tissues.

For autologous cells it is well known that the starting material has wide inherent variability and that the apheresis procedure may impact the actual cell composition and product profile, thus necessitating proper standardization of the donation and collection of the cells [25, 26]. For allogeneic products, there will be limitations on how far material from one donation can be expanded and there will be need for multiple donations from one donor or different donors for large scale production. For different donors the individual variability on the cellular level is usually high, but also cells collected from same donor at different times demonstrate some level of variability. How comparability between products from different donations/donors will be demonstrated remains an open question.

## Balance between Efficacy and Safety

All patients treated with CAR T-cells experience some level of cytokine-release syndrome (CRS), as it is part of the efficacy of the product [27]. CRS is caused by the high activation of T-cells and destruction of numerous tumor cells at the same time (tumor lysis syndrome), both releasing large amounts of cytokines [28]. An especially important role is played by IL-6, which seems to be secreted by monocyte-lineage cells due to the high CAR T-cell activation [29]. Some treated patients have experienced a severe form of CRS, sometimes associated with neurotoxicity and even patient deaths have been reported in many trials, both with autologous and allogeneic CAR T-cells [24, 30]. Severe infections seem also to be frequently reported adverse events (AE) for these products and recently found to be associated with the grade of CRS [31]. Treatment of the severe CRS and neurotoxicity has been focused on blocking the IL-6 with anti-IL-6 antibody tocilizumab, which does not impact the functionality of the CAR T-cells [29]. However, for optimal control of each patient it would be imperative to find predictive biomarkers reflecting the patient characteristics that correlate with efficacy and safety outcomes, and also to identify markers for quality controls that serve the same purpose at the product level. It is already known that severe CRS is associated with high expansion rate of the cells requiring careful consideration of the dose and follow-up of the growth kinetics of the transduced cells [32] and that cytokines used in growth media for stimulation of the cells before transduction have impact on the efficacy outcome [33]. Therefore, monitoring of the potency of the transduced CAR T- cells prior to administration, for example by measurement of antigen-driven *in vitro *cell proliferation, as well as persistence of transduced cells *in vivo*, are important for mitigating these risks.

As emphasized earlier, a key risk factor for CAR T-cells is the specificity of the target antigen, as expression on healthy cells can lead to on-target/off-tumor toxicity and significant damage to patients´ tissues and organs [34]. CD19 is expressed also on healthy B-cells, which can lead to persistent B-cell aplasia and hypogammaglobulinemia in CD19 CAR T –treated patients. This, however, can be managed through replacement therapy and as CD19 is not found on hematopoietic stem cells, the hematopoietic system is not impacted. For solid tumors the available antigen targets may not be adequately tissue specific and approaches to increase specificity may be required, for example by targeting multiple antigens as already explored for B-cell malignancies [35]. Antigen escape of CD19+ and CD22+ tumors has been documented resulting in relapses during the clinical trials [36, 37]. The underlying mechanisms have been studied, yet the results are heterogeneous and demonstrate involvement of alternative splicing (CD19), direct genetic modifications, as well as correlation with pre-existing mutations [36, 37].

Although multi-antigen targeting may represent a suitable strategy to improve on-tumor specificity and to minimize risk of antigen escape, further comparative data is needed to assess the relationship between clinical response profiles of treated CD19- patients and their transduced T-cells. Different factors may interact to influence overall clinical benefit and risk, including: primary sequence and affinity of the antibody domains used for target antigen binding, nature of the co-stimulatory domains, relative level of cell stimulation and activation, tumor burden and the dosing strategy, including pre-conditioning chemotherapy.

The actual cell composition, in terms of the different T-cell subsets, that is administered and expanding in the patient also requires additional understanding. In particular, of interest has been the ratio of CD4+ and CD8+ cells for optimal efficacy [38] and persistence of the CAR T+ subsets [39, 40]; however, there is not yet enough data available to make firm conclusions about the optimal T-cell composition for efficacy and safety. Comparison of published results from clinical trials is hampered by the wide differences in CAR design and actual manufacturing processes. For JCAR017, a specified ratio of CD4+ and CD8+ cells are given to the patients, whereas Kymriah and Yescarta are dosed based on CD3+/CAR T+ numbers (see Table I) [1, 2, 41] . Some data suggest improved persistence of CAR T-cells with the 4-1BB co-stimulatory domain, which seems to support maturation of the CD8+ cells towards central memory phenotype [39, 42]. According to most published trials, persistence of the CAR T-cells is required to prevent relapse after the treatment, although the exact timeframe required is not clear. However, for a CD19 CAR T product with a CD28 co-stimulatory domain it has been reported that persistence of the CAR T cells does not correlate with long term survival [43]. It has been also proposed that CD4+ and CD8+ cells might even require different co-stimulatory domains for their optimal functionality and interplay in eradicating the tumor cells [44]. In addition to the T-cell subsets and the signaling domains of the CAR construct, donor-related factors like age, health status and concomitant medication may also impact the final product and need to be taken into account before manufacturing and dose selection.

Very little discussion can be found on the binding domains of the CAR constructs, yet they comprise an important part of these molecules. Most CD19 CARs utilize the same murine single chain variable fragment (scFv, FMC63), yet for other B-cell targets and for solid tumors humanized or fully human antibody sequences and other binding elements like repeat proteins are utilized [45]. In addition, a human counterpart for FMC63 has been developed and tested in non-clinical models [46]. Human anti-mouse antibodies are known to develop against the FMC63 binding domain, yet these have been considered to have no clinical relevance. However, a severe case of anaphylaxis has been reported resulting from repeated dosing of a CAR T product with murine mesothelin Ab sequence highlighting the need to also consider the origin of the binding domain especially for repeated dosing [47]. Unclear at the moment is whether repeat use of the same virus vectors bears a risk of immune responses against the viral capsids in the treated patients, as inevitably some free virus is transmitted to the patients with the CAR T-cells. This, together with possibility of viral recombination, is the reason why free virus particles in the final product are expected to be analyzed and controlled. Concerns are expressed also about very high affinity of the antibody fragments, as it may lead to responses against cells that have low levels of target expression [45] and it may also reduce the actual anti-tumor activity [48]. On the other hand, the affinity of the scFvs may be reduced over time *in vivo* and careful selection of the binding domain is critical. Studies of same binding domains with varying affinities evaluated side-by-side could be valuable before selection of the final construct for clinical studies, especially in case of target antigens that are expressed also on non-malignant cells.

For autologous products, the best time for cell-based immunotherapies needs to be further investigated. It is well known that tumor burden is related to the severity of the CRS [28], but also the immunological condition of heavily treated patients may be poor and production of sufficient numbers of good quality CAR T-cells may be difficult. New trials are designed to study CAR T-cells together with other immuno-oncology products, like anti-PD1 antagonists [49], which are expected to provide also synergic effects for the CAR T therapy by modifying the tumor inhibitory environment.

## Next Generation

While the first CAR T-cell products are now entering commercial markets, a successful treatment outcome cannot be assured for every patient, and severe adverse events can be expected to occur; CRS may require complex risk management algorithms that are not available in all treatment centers. It should be also recognized how fast the science is evolving in this field, putting the first approved products under heavy pressure as the next generation products will soon follow.

The safety issues have raised the interest towards safety elements (safety switches/suicide genes) that could be introduced into the CARs. For JCAR017, a safety switch composed of a truncated form of epidermal growth factor receptor (EGFR) was introduced into the CAR construct, thus enabling the removal of the transduced cells in emergency situations with anti-EGFR antibody Cetuximab [10] and the RQR8 domain recognized by rituximab is utilized in the allogeneic product UCART19 [4]. In addition, constructs that would allow switching the CAR expression on and off are in non-clinical development, providing more opportunities to control possible toxicities [50].

Traditionally the vectors used for CARs have been retro- or lentiviruses, however, current practice is tending toward lentiviral vectors due to their better safety profile. However, both types of viral vector have potential to integrate into the host genome, thereby raising the concern of insertional oncogenesis (IO) [51]. The problems with integration have been mainly identified when hematopoietic stem cells have been transduced and no IO cases have been reported for genetically modified T-cells yet. However, when more complex products are designed with multiple CAR targets and knock outs of original genes of the cells, the risks are cumulative and can be assessed only when results of long-term safety monitoring become available. For this reason, monitoring treated subjects for secondary malignancies, and collecting biopsy specimens to exclude a causal relationship with insertional mutagenesis, may represent an expected risk management activity. Finally, the trend to use gene editing for CAR T-cell production is raising concerns, as for the technology all issues relating to off-target editing are not yet solved [52]. It is noteworthy that TALEN technology has already been utilized for UCART platform by Cellectis/Servier [25] and Crispr/Cas9 is in preclinical studies for CAR T production [53]. The benefits and risks of the 3rd and 4th generation CAR T-cells, compared to the 2nd generation products, remain to be explored. However, when the intracellular parts are further engineered, the binding domains should not be forgotten and a holistic approach for novel CAR T designs would be valuable, taking into consideration all components that may impact the final outcome in humans.

## Decentralized Manufacturing

Manufacturing of virally transduced cells has raised the need for new manufacturing technologies and devices, especially for autologous products with small amounts of starting materials. Closed systems that allow aseptic sampling have reached the markets providing solutions that can also be applied for individual CAR T manufacturing. Due to the fragility of the transduced cells and short shelf lives, long transportation from the manufacturing facilities may pose challenges to the manufacturers. This has increased the interest towards decentralized manufacturing near the patients and e.g. Miltenyi Biotec has been preparing for this opportunity through the novel devices and by gaining expertise on lentivirus production [54]. However, this scenario leaves the question, who is responsible for the product given to the patient? According to the new guideline on good manufacturing practice (GMP) established for Advanced Therapy Medicinal Products (ATMPs), the products from decentralized manufacturing, if fulfilling the definition of an ATMP, are medicinal products for which the legal requirements apply [55]. Thus, production of an ATMP in a hospital would require same control over the production and over the product, as is defined for industrial manufacturing. The new GMP guideline for ATMPs specifies that Class D background can be accepted for fully closed systems, instead of the standard requirement of Class A in Class B background expected for aseptic production of medicinal products. Here, it must be noted, that Class D background also requires specific production premises with full environmental monitoring (particles and microbes). The production process has to be validated at each site and the product has to be released by a qualified person (QP) against pre-specified and approved specifications. However, the GMP guideline foresees a possibility to establish a “central site” with a QP for oversight of all decentralized sites. This requires written contracts, shared standard operating procedures (SOPs) and understanding of the responsibilities of the QP and production personnel. Considering the complexity of the CAR T-cell products, it remains to be seen, whether decentralized production and central release of autologous cells would be a viable option or whether the allogeneic, off-the-shelf products will conquer the markets.

## Conclusions

The CAR T-cell products have been found to be promising novel therapies for unmet medical need in the oncology sector and the first products are approved for commercial use. Furthermore, hundreds of clinical trials with different CAR T-cells are ongoing and the engineering work of next generation constructs is active. Yet the efficacy results of these products have been profound, all patients do not benefit from the treatment and the number of variables that may impact the clinical outcome of each patient treated with a CAR T product is exceptionally high, both in the autologous and allogeneic approach. In order to mitigate the toxicity and increase number of complete responders, holistic design of novel CAR constructs, as well knowledge of all factors impacting the clinical outcome is needed. In order to gain better understanding of this powerful treatment modality, more data both from patients and product lots need to be collected, so that suitable biomarkers and product quality controls correlating with safety and efficacy can be identified. This link between quality and clinical results is important for establishing limits for potency of the product, but also to control the “living dose” given to patients and mitigate adverse events.

The development of CAR T-cells requires expertise from several areas, including cell and molecular biology, immunology, antibody engineering, risk management, regulatory requirements etc., and collaboration across stakeholders with critical expertise is needed in order to further improve the success of these therapies. CAR T-products are in the frontline of fast evolving science and a product may be already “old” when reaching the markets. Yet, the first products are approved, but true commercial success will also require sustained responses and manageable safety profile in the wider, real world use. On the other hand, heavy manipulation of T-cells and use of recombinant integrating virus vectors may bear unknown risks, which require thorough consideration and mitigation before advancing to clinical studies and extensive follow-up of the patients.

## Abbreviations

Ab: Antibody
AE: Adverse event
ALL: Acute lymphoblastic leukemia
AML: Acute myeloid leukemia
ATMP: Advanced therapy medicinal product
BPDCN: Blastic plasmacytoid dendritic cell neoplasm
CAR: Chimeric antigen receptor
CD: Cluster of differentiation
Crispr: Clustered regularly interspaced short palindromic repeats
CRS: Cytokine release syndrome
DLBCL: Diffuse large B-cell lymphoma
DSMB: Data Safety Monitoring Board
EGFR: Epidermal growth factor receptor
EU: European Union
FDA: Food and Drug Administration
GE: Gene editing
GMP: Good manufacturing practise
GvHD: Graft vs. Host Disease
HLA: Human leucocyte antigen
IO: Insertional oncogenesis
ko: Knock out
MA: Marketing authorisation
MM: Multiple myeloma
NK cell: Natural killer cell
PBMC: Peripheral blood mononuclear cells
QP: Qualified person
R/R: Relapsed/refractory
scFv: Single chain variable fragment
SOP: Standard operating procedure
TAA: Tumor associated antigen
T-cell: T lymphocyte
TRUCK: T cells Redirected for Universal Cytokine-mediated Killing
US: United States
